# Mesenteroaxial volvulus in an adult: time is of the essence in acute presentation

**DOI:** 10.2349/biij.5.3.e18

**Published:** 2009-07-01

**Authors:** S Singham, B Sounness

**Affiliations:** Department of Medical Imaging, John Hunter Hospital, Newcastle, Australia

**Keywords:** Mesenteroaxial volvulus, paraoesophageal herniae

## Abstract

Acute gastric volvulus is an uncommon condition with severe repercussions if untreated in the acute presentation. We describe such a case. We assert that computed tomography (CT) should be the first line of investigation

## CASE REPORT

81 year-old female with known paraoesophageal herniae presented with an acute episode of haematemesis and severe epigastric pain. The patient had a background of hypertension, peptic ulcer disease and gastro-oesophageal reflux disorder.

**Figure 1:**
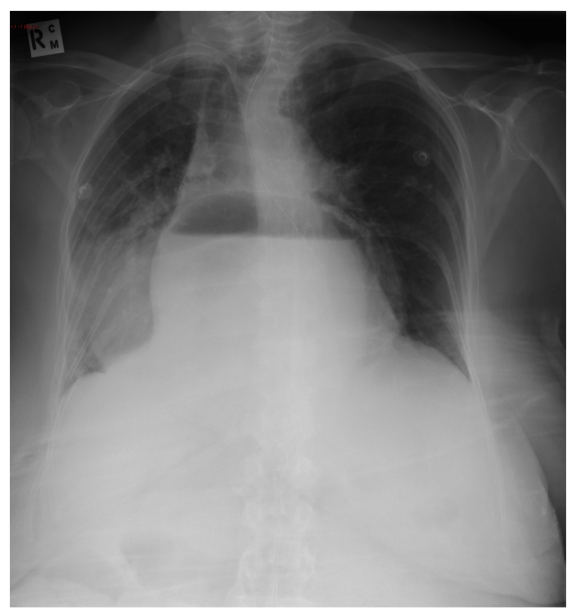
CXR demonstrates large hiatus herniae with air-fluid level projected over the heart. Nasogastric tube can be seen at approximately T9 level.

Gastroscopy was undertaken, confirming large paraoesophageal herniae. A CXR showed large hiatus herniae (Figure 1). The patient remained in the ward and deteriorated acutely. The herniae was thought to be incarcerated and laparotomy with repair of paraoesophageal herniae was planned. Unfortunately, the patient became unconscious, was unresponsive to resuscitative efforts and passed away.

**Figure 2:**
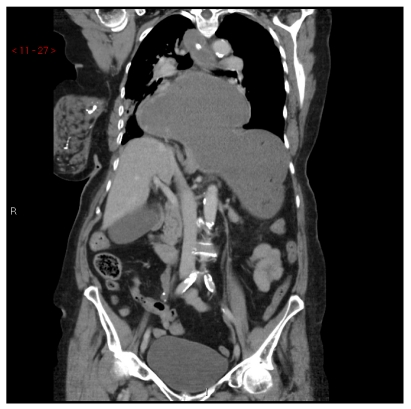
Part of the body and antrum of the stomach are demonstrated above the diaphragm. The fundus is located below the diaphragm. The duodenum is compressed against the diaphragm.

CT demonstrated complex hiatus hernia (Figure 2). The antrum and part of the body of the stomach as well as proximal duodenum was above the diaphragm. The oesophagus and fundus as well as the remaining part of the body of the stomach was located below the diaphragm. The stomach was markedly distended and the duodenum appeared to be compressed at the level of the diaphragm by the stomach.

**Figure 3:**
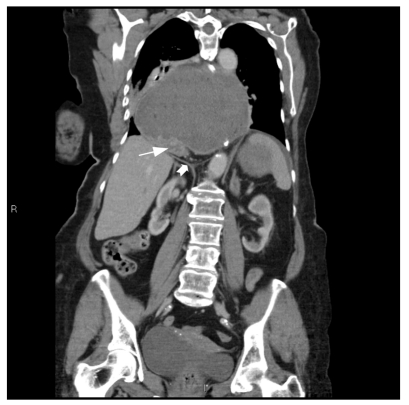
Coronal CT image demonstrates the duodenum compressed against the diaphragm. The distal body and antrum are distended with fluid and superior to the diaphragm. (White arrow: duodenum, Fat white arrow: diaphragm)

**Figure 4:**
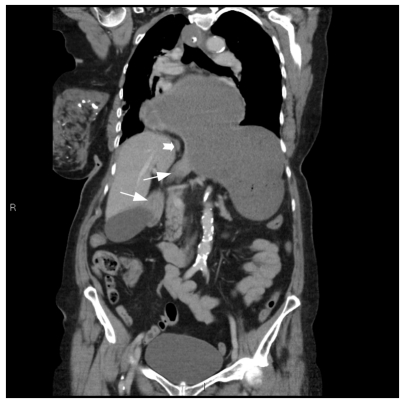
More anteriorly the duodenum is seen curling around the diaphragm and entering the abdomen via the oesophageal hiatus. The proximal body and fundus are seen below the diaphragm. (White arrows: duodenum, thick white arrow: diaphragm at the hiatus)

**Figure 5:**
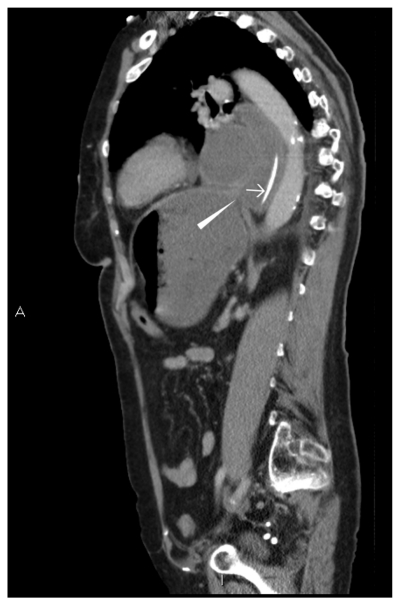
Left sagittal image demonstrates an abrupt narrowing of the fluid-filled oesophagus at the level of the diaphragm. The nasogastric tube is demonstrated unable to pass into the stomach. (White arrowhead: oesophageal narrowing at diaphragm, White arrow: nasogastric tube)

## DISCUSSION

Acute gastric volvulus usually presents with Borchardt triad of epigastric pain, retching without vomiting, and inability to pass nasogastric tube (due to distortion of the anatomy at the gastroesophageal junction) [1].

Gastric volvulus is defined as an abnormal rotation of the stomach of more than 180 degrees, creating a closed loop obstruction. According to the axis around which the stomach rotates it may either be organoaxial or mesenteroaxial, or a combination of both [2].

Mesenteroaxial volvulus (which is the less common variant - 29% of cases [3]) is where the stomach rotates around a transverse axis connecting the middle of the greater and lesser curvatures. Gastric volvulus can occur at any age, however, it is more common in children [4] with equal frequency in both men and women [2].

Clinically, gastric volvulus can present as either an acute abdominal emergency or as recurrent volvulus [5]. Intra abdominal gastric volvulus is usually associated with contributing anatomic factors: abnormal stomach mobility due to a lack of, or markedly lax ligaments, gastric tumour, splenic or left hepatic lobe agenesis [6]. Prompt recognition and decompression are required to prevent infarction and perforation [6].

Traditionally acute gastric volvulus is diagnosed on a chest X-ray showing retrocardiac air bubble or large air-fluid level in the chest [7]. A contrast study showing obstruction of the stomach at the site of the volvulus confirms this diagnosis [7]. However, a CT scan can offer an immediate diagnosis with anatomical details. Coulier and Ramboux [7] assert that helical CT should be the first choice technique of imaging as it avoids any delay in diagnosis. The CT and MR appearance may be variable depending on the extent of gastric herniation and the point of torsion of the stomach. In mesenteroaxial volvulus, CT may show gastric herniation of the antrum and distal body in the left hemithorax, with inferior location of the oesophagogastric junction below the diaphragm [8].

Coulier and Ramboux further assert that the frequency of this disease is probably underestimated because of the existence of partial and/or spontaneously reversible forms [7].

In the case of paraoesophageal herniation, radiological examination is again diagnostic. Films of the chest or abdomen may demonstrate a high “stimulated” left diaphragm which is actually the herniated and distended greater curvature of the stomach [9]. Double air fluid levels occur if the fundic portion of the thoracic stomach redescends into the abdomen. There may be a “hairpin” loop with the incisura directed toward the right upper quadrant or posteriorly [9]. Massive gaseous distension in the upper abdomen or chest may appear, with a gas bubble on either side of the midline [9]. Barium swallow studies demonstrate the classic signs of volvulus, such as sharp cut-off of the oesophageal or gastric barium column, abnormal twisting of the rugal folds, and finally delineation of the intrathoracic portion of the stomach [9].

The treatment is surgical consisting of laparotomy, de-rotation and internal fixation [6].Gangrenous portions are resected. Recurrent volvulus should be prevented by anterior gastropexy where the greater curvature of the stomach is fixed to the anterior abdominal wall [5], and repair of the diaphragmatic defect should be undertaken [8].
